# Molecular testing for the clinical diagnosis of fibrolamellar carcinoma

**DOI:** 10.1038/modpathol.2017.103

**Published:** 2017-09-01

**Authors:** Rondell P Graham, Matthew M Yeh, Dora Lam-Himlin, Lewis R Roberts, Luigi Terracciano, Michael W Cruise, Patricia T Greipp, Riyam T Zreik, Dhanpat Jain, Nida Zaid, Safia N Salaria, Long Jin, Xiaoke Wang, Jeanette G Rustin, Sarah E Kerr, William R Sukov, David A Solomon, Sanjay Kakar, Emily Waterhouse, Ryan M Gill, Linda Ferrell, Venancio AF Alves, Deniz Nart, Funda Yilmaz, Stephanie Roessler, Thomas Longerich, Peter Schirmacher, Michael S Torbenson

**Affiliations:** 1 Department of Laboratory Medicine and Pathology, Mayo Clinic, Rochester, MN, USA; 2 Department of Pathology, University of Washington, Seattle, WA, USA; 3 Department of Laboratory Medicine and Pathology, Mayo Clinic, Scottsdale, AZ, USA; 4 Division of Molecular Pathology, Institute of Pathology, University Hospital Basel, Basel, Switzerland; 5 Department of Pathology, Cleveland Clinic, Cleveland, OH, USA; 6 Department of Pathology, Baylor Scott & White Memorial Hospital, Temple, TX, USA; 7 Department of Pathology, Yale, New Haven, CT, USA; 8 Department of Pathology, Microbiology and Immunology, Vanderbilt University, Nashville, TN, USA; 9 Department of Pathology, University of California San Francisco, San Francisco, CA, USA; 10 Instituto do Câncer do Estado de São Paulo, Faculdade de Medicina da Universidade de, São Paulo, Brazil; 11 Department of Pathology, Ege University, Izmir, Turkey; 12 Institute of Pathology, University Hospital, Heidelberg, Germany; 13 Institute of Pathology, University Hospital RWTH, Aachen, Germany

## Abstract

Fibrolamellar carcinoma has a distinctive morphology and immunophenotype, including cytokeratin 7 and CD68 co-expression. Despite the distinct findings, accurate diagnosis of fibrolamellar carcinoma continues to be a challenge. Recently, fibrolamellar carcinomas were found to harbor a characteristic somatic gene fusion, *DNAJB1–PRKACA*. A break-apart fluorescence *in situ* hybridization (FISH) assay was designed to detect this fusion event and to examine its diagnostic performance in a large, multicenter, multinational study. Cases initially classified as fibrolamellar carcinoma based on histological features were reviewed from 124 patients. Upon central review, 104 of the 124 cases were classified histologically as typical of fibrolamellar carcinoma, 12 cases as ‘possible fibrolamellar carcinoma’ and 8 cases as ‘unlikely to be fibrolamellar carcinoma’. *PRKACA* FISH was positive for rearrangement in 102 of 103 (99%) typical fibrolamellar carcinomas, 9 of 12 ‘possible fibrolamellar carcinomas’ and 0 of 8 cases ‘unlikely to be fibrolamellar carcinomas’. Within the morphologically typical group of fibrolamellar carcinomas, two tumors with unusual FISH patterns were also identified. Both cases had the fusion gene *DNAJB1–PRKACA*, but one also had amplification of the fusion gene and one had heterozygous deletion of the normal *PRKACA* locus. In addition, 88 conventional hepatocellular carcinomas were evaluated with *PRKACA* FISH and all were negative. These findings demonstrate that FISH for the *PRKACA* rearrangement is a clinically useful tool to confirm the diagnosis of fibrolamellar carcinoma, with high sensitivity and specificity. A diagnosis of fibrolamellar carcinoma is more accurate when based on morphology plus confirmatory testing than when based on morphology alone.

Fibrolamellar carcinoma is a rare primary liver carcinoma with distinctive clinical and morphologic characteristics. Fibrolamellar carcinoma is not associated with elevated serum alpha fetoprotein levels, is enriched in younger age groups, and is not associated with underlying liver disease. The tumor is characterized by neoplastic cells with abundant eosinophilic granular cytoplasm, prominent nucleoli, and striking intratumoral fibrosis, classically arranged in parallel or lamellar bands. At the immunohistochemical level, fibrolamellar carcinoma is characterized by cytokeratin 7^1, 2^ and CD68 co-expression.^3^ These immunostains are useful to support the diagnosis of fibrolamellar carcinoma.

Despite the distinctive histologic and clinical features of fibrolamellar carcinoma, misclassification is a persistent problem, most commonly with cases of conventional hepatocellular carcinoma incorrectly classified as fibrolamellar carcinoma. As one example, data from the SEER database found an average age of 39 years for fibrolamellar carcinoma,^4^ which is significantly older than the average age of 27 found in pathologically confirmed cases reported in original studies.^5^ Other studies have specifically examined primary liver tumors with abundant intratumoral fibrosis and found that they can closely mimic fibrolamellar carcinoma.^6^ These findings suggest the need for more objective diagnostic markers for fibrolamellar carcinoma. Proper classification of tumors is the foundation on which modern clinical management and therapy is based. In this regard, the likelihood of significant advancements in understanding the biology of fibrolamellar carcinoma and developing novel therapies depends in the first place on studying cases that are actually fibrolamellar carcinomas.

Honeyman *et al* discovered a novel somatic recurrent 400 kb deletion on the short arm of chromosome 19, giving rise to an in-frame *DNAJB1–PRKACA* gene fusion in fibrolamellar carcinoma.^7^ The resulting fusion protein is thought to constitutively activate the kinase activity of protein kinase A catalytic subunit alpha and to function as the oncogenic driver of fibrolamellar carcinoma. Therefore, its detection provides a robust diagnostic biomarker. A subsequent study found the fusion transcript in only ~80% of fibrolamellar carcinomas, but most of the cases in this study did not undergo central pathology review.^8^ Recently, a clinical test for fibrolamellar carcinoma has been developed based on fluorescent *in situ* hybridization (FISH) to detect the *PRKACA* rearrangement and was positive in all of 26 cases of fibrolamellar carcinoma and in none of the conventional hepatocellular carcinomas.^9^

Currently, fibrolamellar carcinoma is diagnosed in most centers based on H&E morphology, often supplemented with immunostains for CK7 and CD68. In order to further validate FISH based testing for routine clinical care, we undertook a retrospective multi-institutional, multinational study of a large number of cases originally diagnosed as fibrolamellar carcinoma based on morphology. The primary goal of this study is to examine the diagnostic utility of the *PRKACA* FISH assay by examining a larger cohort of clinical specimens including resections, needle biopsies, and cytology aspirates. As part of this, in a subset of cases the FISH test was applied to multiple sections from the same tumor as well as to cases with primary and metastatic disease. The second goal was to identify fibrolamellar carcinoma cases with unusual FISH patterns, which may provide novel biological insights and represent interpretative challenges in clinical diagnosis.

## Materials and methods

**Figure 1:**
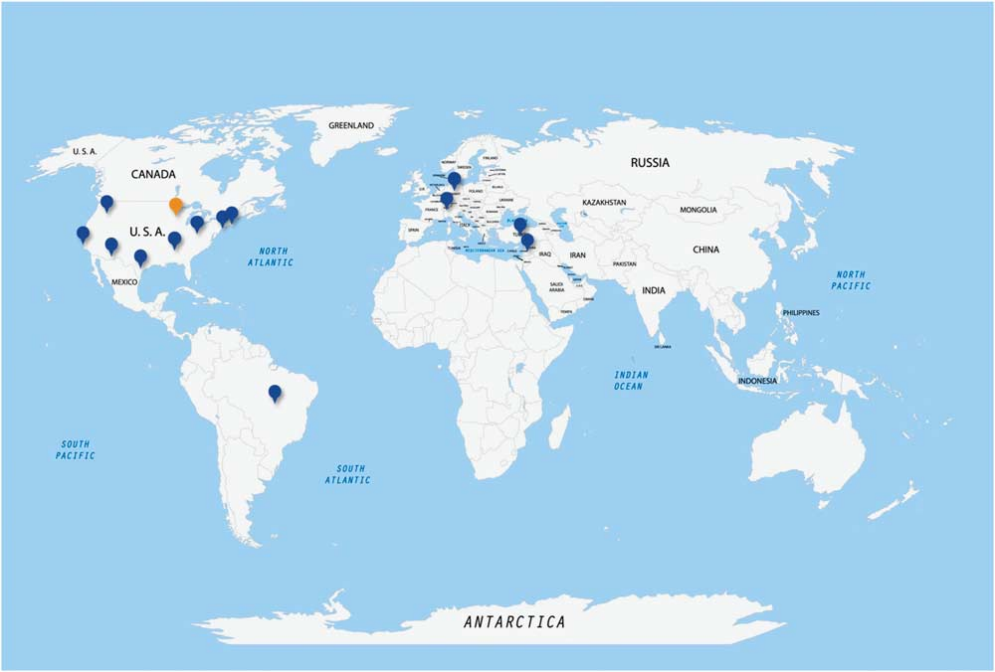
Fibrolamellar carcinoma origins—The geographic origin of the cases of fibrolamellar carcinoma included in this study.

We retrospectively retrieved cases diagnosed as fibrolamellar carcinoma from the institutional archives or consultation files of numerous medical centers from around the world (Figure 1). Slides and tissue blocks were retrieved from patients who provided informed consent per required institutional protocols. The study was approved by the institutional review boards of the respective institutions and in concordance with ethical guidelines of the 1975 Declaration of Helsinki.

All of the retrieved cases were centrally reviewed simultaneously by two of the authors (RPG and MST) blinded to clinical information, the results of immunohistochemistry, and FISH results. The study cases include 112 resections and 12 biopsies. The formalin fixed paraffin embedded tissue cases were classified based on morphology alone into typical examples of fibrolamellar carcinoma, cases which possibly represented fibrolamellar carcinoma, and cases which were unlikely to be fibrolamellar carcinoma. The criteria used for typical fibrolamellar carcinoma included the presence of the following morphological findings: (i) eosinophilic, monotonous neoplastic cells with (ii) abundant granular cytoplasm, (iii) open nuclear chromatin, (iv) conspicuous macronucleoli, and (v) dense and/or lamellar type intratumoral fibrosis. Alcohol-fixed smears from six cases associated with core biopsy specimens were also reviewed (RPG and SEK). Unstained sections from a tissue microarray composed of 88 conventional hepatocellular carcinoma, 6 cases of fatty liver disease, and 7 examples of normal liver (SR, TL, and PS) along with unstained sections from a tissue microarray with eight fibrolamellar carcinomas (VA) were obtained for FISH.

Immunohistochemistry for CK7 (clone OV-TL; Dako) and CD68 (clone KP1; Dako) were performed at the Mayo Clinic on unstained slides of full tissue sections from each of the cases diagnosed as fibrolamellar carcinoma.^10^ FISH was performed and interpreted using a previously published break-apart probe set for the *PRKACA* locus.^9^ Because fusion genes in cancer often have multiple partners, we used a break-apart probe strategy for the FISH. In this assay, a green and a red probe target adjacent sequences of DNA. The green probe binds to the PRKACA gene while the red probe binds adjacent and will be lost regardless of the fusion partner. Because the two probes are adjacent in normal liver tissue, two yellow signals are seen in cells without the deletion, one for each chromosome. However, the 400 kb deletion seen in fibrolamellar carcinoma results in loss of the DNA covered by the red probe. Thus, a positive result is typically a separate green signal, (loss of a single red signal) with a solitary intact yellow fusion signal, per tumor nucleus. To be considered positive, the abnormal signal pattern had to be present in greater than 50% of the tumor cells. This cutoff was empirically determined using examples of normal liver tissue, of which no example ever had more than 5% of cell nuclei had any abnormal pattern.

After histologic review, immunohistochemistry, and FISH results, patient ages at diagnosis and history of background liver disease were reviewed.

To investigate further one of the cases with amplification of the FISH probes, quantitative PCR (qPCR) for *PRKACA* exon 8 and 9 (encoding the kinase domain) was performed on four randomly selected fibrolamellar carcinomas, a single randomly selected cholangiocarcinoma, and a single case of fibrolamellar carcinoma with an unusual FISH result. This assay measures total PRKACA RNA and does not specifically identify the fusion transcript. Total RNA was extracted from formalin fixed paraffin embedded tissues using miRNeasy FFPE Kit (Qiagen, CA) and was treated with DNase I (Life Technologies). RNA concentration was measured by Qubit 2.0 Fluorometer (Thermo Fisher Scientific, MA). cDNA was synthesized with random hexamers and 1 μg of total RNA using the iScript cDNA Synthesis kit (Bio-Rad, CA). qPCR was performed on a LightCycler 480instrument (Roche, Basel, Switzerland) with 96-well plates. Human Universal Reference cDNA (Takara Bio, CA) was used as the calibrator. Hydrolysis probe UPL # 6 (Roche, Basel, Switzerland) and PCR primer set (*PRKACA*-qPCR-F, 5′-TTTGCCACAACTGACTGGAT-3′ *PRKACA*-qPCR-R, 5′-CCAGGGCCTTTAAACTTTGG-3′) were used to amplify a 77 bp amplicon of *PRKACA* mRNA. Hydrolysis probe UPL # 69 (Roche, Basel, Switzerland) and PCR primer set (*PGK1*-qPCR-F, 5′-GGAGAACCTCCGCTTTCAT-3′ *PGK1*-qPCR-R, 5′-GCTGGCTCGGCTTTAACC-3′) were used to amplify a 78 bp amplicon of the reference *PGK1* mRNA. Each 20 μl qPCR assay included 1 × BiolineSensiFAST Probe No-ROX Mix (Bioline, MA), 6 uM of each primer, and 3 uM of probe and 5.0 μl of diluted cDNA. qPCR conditions are as follows: incubation at 95 °C for 4 min followed by 45 cycles of 95 °C for 10 s and 60 °C for 50 s. Relative expression ratios were calculated using the Calibrator Normalized Relative Quantification Method (Roche, Basel, Switzerland). qPCR for the *PRKACA* target gene and the reference *PGK1* gene were carried out in triplicate wells, respectively.

Finally, tumor DNA from a single fibrolamellar carcinoma case with atypical FISH results was evaluated with a SNP array (OncoScan, Affymetrix) following the vendor provided instructions.

## Results

We gathered cases from 124 patients that were diagnosed as fibrolamellar carcinoma. In 17 of these cases, there were 2 or 3 additional samples available from a single patient for analysis, including 6 cases with alcohol-fixed cytology preparations and paired formalin fixed paraffin embedded tissue sections. In the 17 cases with multiple samples, each of the samples from separate time points or the same procedure showed concordant results. Forty-two (34%) cases were from the Mayo Clinic in Rochester or the consultations files of the senior author (MST). Various clinical and biological aspects were previously published, including FISH results, on twenty-eight cases from the Mayo Clinic, Cleveland Clinic, and the University Hospital of Basel.^9, 10, 11^

Based solely on morphology, the tumors were classified at central review as histologically typical of fibrolamellar carcinoma in 104 (84%) cases. In 12 (10%) cases, the histology was classified as possible fibrolamellar carcinoma and in 8 (6%) cases the tumor was classified as unlikely to be fibrolamellar carcinoma. The results are discussed below using the central review histological classification of typical, possible, or unlikely to be fibrolamellar carcinoma.

### Typical Fibrolamellar Carcinoma

Patient ages were available in 85 of 104 (82%) cases. The average was 23.8±7.8 years for patients with tumors classified as typical fibrolamellar carcinoma. The cases showed the characteristic morphology of fibrolamellar carcinoma. In four cases, the tumors showed areas of solid growth without intratumoral fibrosis. However, typical areas were present elsewhere in these cases. Each of the 6 cytology specimens was classified as typical for fibrolamellar carcinoma.

Immunohistochemistry was performed in 92 of 104 typical fibrolamellar carcinoma cases. Cytokeratin 7 was diffusely positive in 87 cases (95%), patchy in 2 cases (2%), focally expressed in 2 cases (2%), and negative in a single case (1%). CD68 was diffusely positive in 83 (90%) cases, patchy in 2 (2%), weak in 5 (5%), and negative in 2 (2%) cases. In 1 case, CK7 and CD68 were both negative. The negative staining cases all had questionable staining quality, showing a lack of staining of the internal controls (bile ducts for CK7, Kupffer cells/macrophages for CD68) or were very small biopsies with limited tissue (1 case).

**Figure 2:**
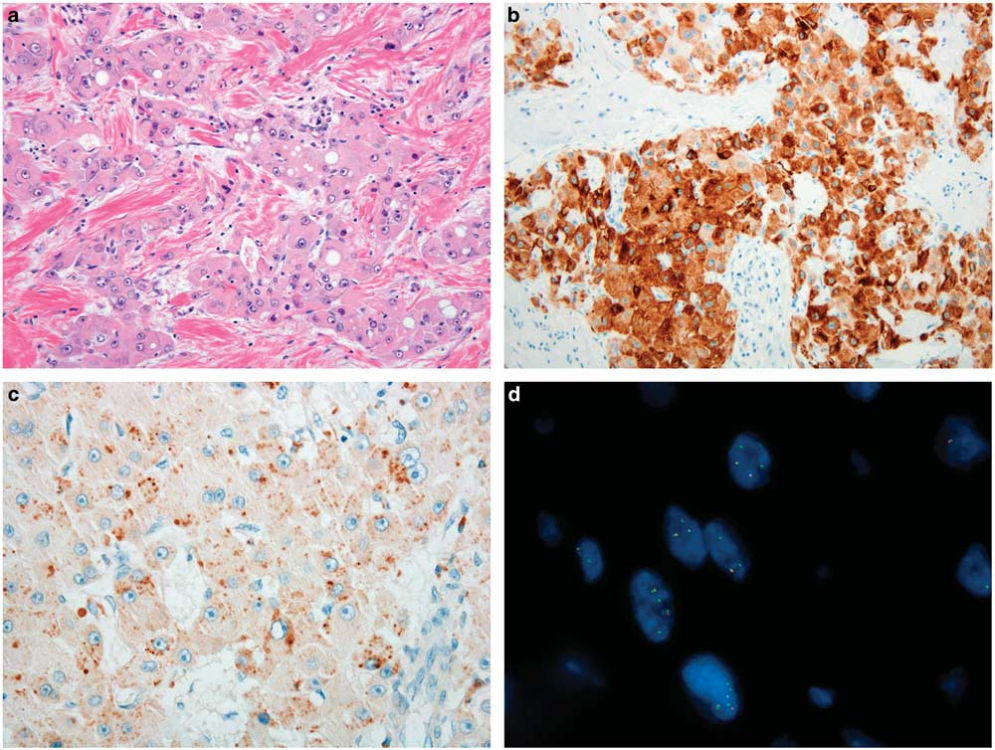
Typical fibrolamellar carcinoma results. (**a**) Typical fibrolamellar carcinoma. The neoplastic cells are characterized by abundant eosinophilic cytoplasm, nuclei with open chromatin, and prominent nucleoli. The tumor cells form trabecula which are separated by bands of fibrosis. (**b**) Cytokeratin 7 is positive in the tumor cells. (**c**) A CD68 immunostain shows characteristic cytoplasmic staining. (**d**) *PRKACA* break apart FISH in a typical fibrolamellar carcinoma. The FISH result is positive. The tumor cells show separate green signals and intact yellow signals (due to overlapping red and green signals).

*PRKACA* FISH was successful in all but 1 case (*n*=103 of 104). *PRKACA* was rearranged in 102 of 103 cases tested. The areas with sheet like growth (seen in four cases) showed the same FISH pattern as areas with the classical growth pattern. FISH was successful in 4 of 6 alcohol-fixed cytology preparations, with two cases failing to hybridize. Each of the 4 alcohol-fixed cytology specimens were positive for *PRKACA* rearrangement. Figure 2 shows an example of typical fibrolamellar carcinoma, its immunophenotype and *PRKACA* FISH result. The most common rearrangement pattern showed a separate green signal with an intact yellow signal per nucleus, followed by two green signals and two intact yellow signals per nucleus. These patterns were seen in 100 of 102 (98%) cases. In the remaining two cases there were unusual signal patterns that were still consistent with *PRKACA* rearrangement, but also showed additional FISH abnormalities.

**Figure 3:**
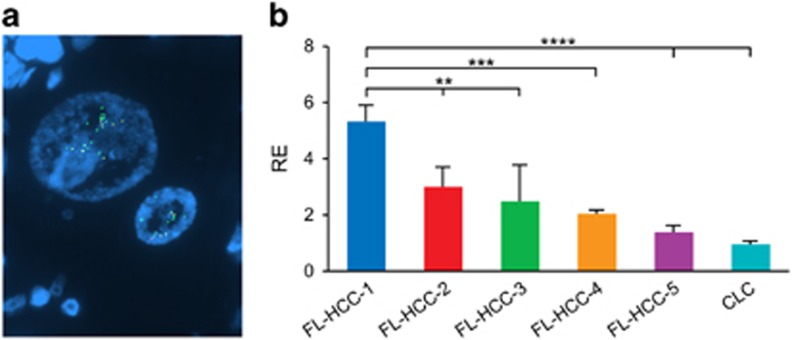
Fibrolamellar carcinoma with PRKACA locus amplification. (**a**) *PRKACA* break Apart FISH results are consistent with amplification of the *PRKACA* locus. The fibrolamellar carcinoma showed typical morphology. There are greater than 10 separate green signals per tumor cell nucleus with fewer intact yellow signals. (**b**) Quantitative reverse transcription-PCR shows a 2–5-fold increase in *PRKACA* mRNA compared to four randomly selected fibrolamellar carcinomas (FL-HCC-2-5) and one randomly selected cholangiocarcinoma (CLC).

In the first case with unusual FISH results, there were >10 separate green signals per nucleus, with only 1 or 2 intact yellow signals per nucleus, a finding seen in ~30% of the tumor nuclei (Figure 3). These results suggest amplification of the rearranged *PRKACA* locus. Follow-up qPCR confirmed a 2–5-fold increase in *PRKACA* mRNA in this case, compared to 4 randomly selected fibrolamellar carcinoma cases with typical FISH patterns (Figure 3).

**Figure 4:**
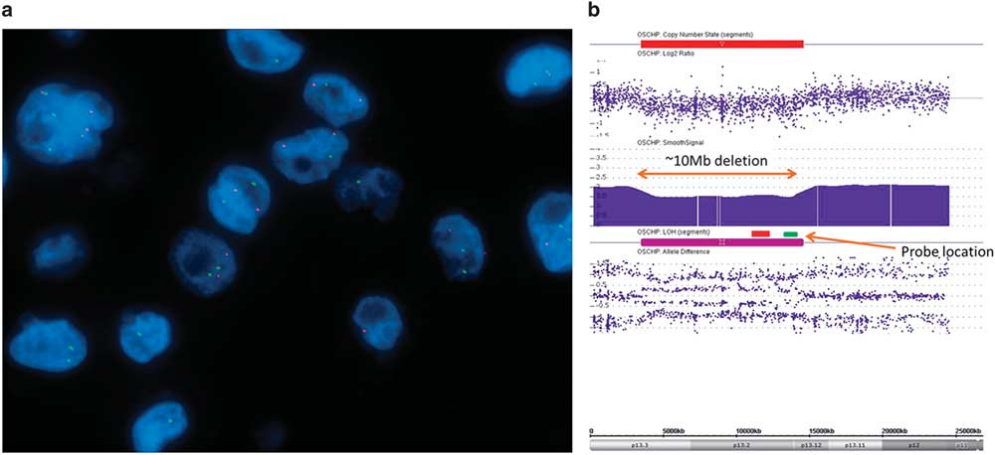
Fibrolamellar carcinoma with uncommon FISH results (**a**) *PRKACA* break-apart FISH shows separate red and green signals in a morphologically typical fibrolamellar carcinoma. This FISH signal pattern raised the possibility of either a different fusion partner for *PRKACA* or heterozygous loss of the *PRKACA* locus without involvement in a gene fusion event. (**b**). Array comparative genomic hybridization data at chromosome 19p demonstrated heterozygous loss of the PRKACA locus due to a 10 Mb deletion (orange bidirectional arrow) including the hybridization site of the FISH probe (shown in green).

The second unusual FISH rearrangement pattern showed separate single red and single green signals per nucleus, without an intact yellow signal. We performed RT-PCR for *DNAJB1–PRKACA* with two different primer sets and confirmed the presence of the *DNAJB1–PRKACA* fusion transcript (Figure 4). We then followed up with a SNP array and identified a 10MB heterozygous deletion which encompassed the hybridization site for the green probe of the *PRKACA* probe set (Figure 4). Taken together, these data are consistent with deletion of the intact *PRKACA* locus on one allele of chromosome 19, with formation of the *DNAJB1–PRKACA* fusion gene on the other allele.

The single typical fibrolamellar carcinoma without a *PRKACA* rearrangement by FISH affected a 14-year-old girl with a family history of the Carney Complex.^12^

### Possible Fibrolamellar Carcinoma

**Figure 5:**
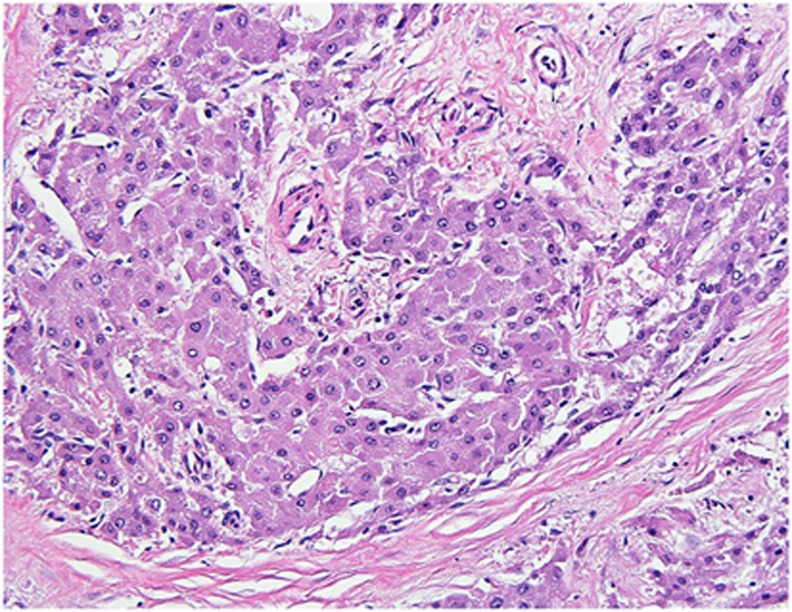
Possible fibrolamellar carcinoma. An example of a case classified as a ‘possible fibrolamellar carcinoma’ based on morphology, characterized by intratumoral fibrosis and neoplastic cells with granular cytoplasm. The cytoplasm of the tumor cells is amphophilic in nature. This case was positive for PRKACA break-apart by FISH testing.

Cases were classified histologically as possible fibrolamellar carcinoma when they showed some but not all of the typical features of fibrolamellar carcinoma (Figure 5). Immunohistochemistry showed diffuse CK 7 staining in 7/12 cases, patchy staining in 2/12 cases and no staining in 3/12 cases. CD68 was expressed in 11 cases and negative in a single case. The CD68-negative case was cytokeratin 7 positive. FISH was successful in all 12 cases and was positive for *PRKACA* rearrangement in 9 cases, of which 8 showed the typical co-expression of cytokeratin 7 and CD68, while the last case was cytokeratin 7 positive but CD68 negative. In each of the 9 cases where *PRKACA* was rearranged, the FISH signal pattern was characteristic (1 green and 1 yellow signal per nucleus). The three FISH negative cases were re-classified as not fibrolamellar carcinoma.

### Unlikely Fibrolamellar Carcinoma

**Figure 6:**
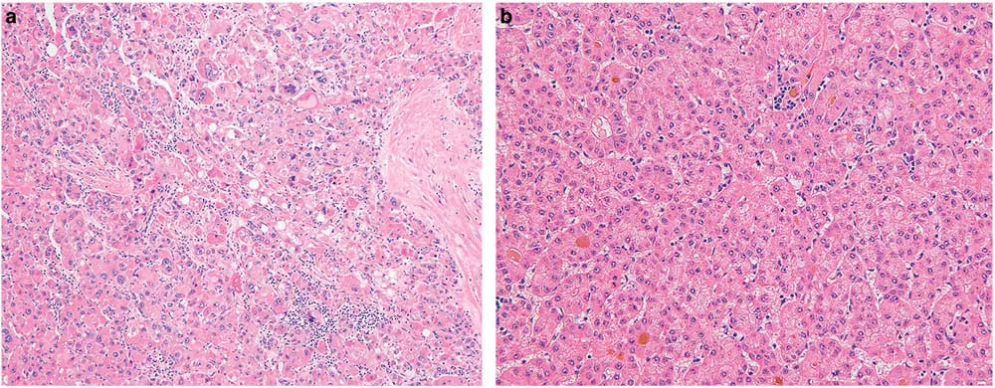
Unlikely fibrolamellar carcinoma An example of a case classified as ‘unlikely to be fibrolamellar carcinoma’ based on morphology. (**a**) The tumor shows more nuclear pleomorphism than is seen in typical fibrolamellar carcinomas. (**b**) The growth patterns shows pseudoacinar areas with cholestasis. This case was negative for PRKACA break-apart by FISH testing.

The cases classified as unlikely to be fibrolamellar carcinoma had some morphological features to suggest a diagnosis of fibrolamellar carcinoma but also had findings that made the diagnosis less likely, such as significant nuclear pleomorphism or the lack of distinct macronucleoli or abundant eosinophilic cytoplasmic (Figure 6). Immunohistochemistry was completed on 7 of 8 cases classified as ‘unlikely fibrolamellar carcinoma’. Cytokeratin 7 was diffusely positive in 3 cases, patchy in 2 others, and negative in the remaining 2 cases. CD68 was positive in 1 case and negative in the other 6 cases. One case showed cytokeratin 7 (patchy) and CD68 co-expression. FISH for *PRKACA* rearrangement was negative in all cases, showing two intact *PRKACA* loci (Figure 6).

All of the cases classified as unlikely to be fibrolamellar carcinoma based on central H&E review were finally classified as conventional hepatocellular carcinoma. Clinical data were available for 7 of these cases. The ages at resection ranged from 47 to 72 years, with a median of 61 years. Two of these individuals had histories of chronic hepatitis B. One case had portal fibrosis in the background liver, while two had cirrhosis.

### Tissue Microarrays

On the first tissue microarray, all of the 88 conventional hepatocellular carcinomas were negative for *PRKACA* rearrangements, as were samples of non-neoplastic liver that were either normal (*N*=7) or showed fatty change (*N*=6). Each of the eight (100%) fibrolamellar carcinomas in the second tissue microarray was positive for *PRKACA* rearrangement in the typical pattern. These eight cases were not included in the calculations done in this study because whole-slide sections were not reviewed, but serve as a positive control for the tissue microarrays of conventional hepatocellular carcinoma, ensuring that the FISH assay works well on microarray samples.

## Discussion

The results from this study demonstrate the utility of FISH as a clinical test to detect the *DNAJB1–PRKACA* that is found in fibrolamellar carcinomas. The FISH test is highly sensitive and specific, as shown in this multicenter study of over 100 cases from North America, South America, and Europe. Additional tests that can confirm a diagnosis of fibrolamellar carcinoma are important. While the morphological findings are distinctive, there can be overlap with conventional hepatocellular carcinomas, as demonstrated by the results of this study, where 9% of cases submitted as fibrolamellar carcinomas could not be confirmed, a problem that is also extensively discussed in review articles.^5, 13, 14^ As a result of this problem, many published studies of fibrolamellar carcinoma appear to include tumors that are most likely not fibrolamellar carcinomas, but rather other types of hepatocellular carcinoma. This outcome is suboptimal, as advances in understanding the biology and potential therapies for fibrolamellar carcinoma are most likely to occur when studies exclusively examine fibrolamellar carcinomas. Attempts to address this problem began with the report of overexpression of anterior gradient-2 protein as a marker of fibrolamellar carcinoma^15^ and continued with studies showing the utility of CD68 and cytokeratin 7 co-expression in confirming a histological diagnosis of fibrolamellar carcinoma.^3^ While immunostains for CK7 and CD68 are widely available and easy to use, the CD68 stain in particular can fail in some cases for technical reasons. The seminal discovery of the *DNAJB1–PRKACA* fusion^7^ has permitted the development of the next generation of testing.^9^ This molecular based testing is robust and has the advantage of identifying the defining genetic lesion of this tumor. We propose that the diagnosis of fibrolamellar carcinoma should now be based on (1) compatible morphology and (2) confirmatory testing, with preference for molecular testing. When molecular testing is not available, co-expression of CK7 and CD68 would be an acceptable alternative. This approach is well in line with the generally accepted method for defining hepatocellular carcinoma morphological subtypes.^16^ A strict definition will also better serve patients when enrolling in clinical trials for the treatment of fibrolamellar carcinoma. The use of confirmatory testing does not impugn the diagnostic skills of pathologists, who still need to identify cases needing testing based on morphology, but instead reflects the well documented need for confirmatory testing to ensure correct diagnosis. This approach allows patients an early benefit from the molecular revolution and will hopefully lay the foundation for future advances.

The data in this study shows that the detection of *PRKACA* rearrangement has a sensitivity of 99% for the diagnosis of fibrolamellar carcinoma. The test performs well on biopsies and can even be extended to alcohol-fixed cytology specimens.

All but one case of typical fibrolamellar carcinoma was positive for *PRKACA* rearrangement. The diagnosis for this case was confirmed by the combination of characteristic morphology and characteristic immunohistochemistry for cytokeratin 7 and CD68. While neither morphology nor immunohistochemistry are infallible, in this case, their combined use in experienced hands provides strong support for the diagnosis. The clinical information (age, absence of background liver disease, family history of the Carney complex in first degree relative) and the current genetic model for fibrolamellar carcinoma are all in keeping with that diagnosis for this case. This case affected a 14-year-old female with a history of the Carney complex in her mother.^12^ The Carney complex is characterized by germ line *PRKAR1A* mutations in the majority of patients.^17^ The model of fibrolamellar carcinogenesis as a tumor driven by activation of protein kinase A would be fulfilled in this patient’s case by loss of *PRKAR1A* function, which encodes a negative regulator of protein kinase A activity. The combination of a germ line hit and a second somatic hit would allow for over activity of the catalytic subunit PRKACA. This hypothesis of course requires confirmation.

The results from this study significantly extend the number of non-fibrolamellar carcinoma cases tested for *DNAJB1–PRKACA* fusion (*N*=88). All of these cases were negative for the *DNAJB1–PRKACA* fusion. In addition, two other studies have evaluated a combined 87 cases of non-fibrolamellar hepatocellular neoplasms for *DNAJB1–PRKACA*^9, 18^ and all were negative. These finding together (*N*=175 total cases) indicate that the *DNAJB1–PRKACA* fusion is highly specific for fibrolamellar carcinoma.

The results from this study also show that the *DNAJB1–PRKACA* fusion is consistently detected in multiple samples of different tumors (primary, metastases) from the same patient. In addition, the fusion is present throughout any given tumor, even when there are varied growth patterns, including areas of solid growth that lack the striking intramural fibrosis, areas that have sometimes led to classification of such cases as mixed hepatocellular carcinoma and fibrolamellar carcinoma. All of these findings support the concept that the *DNAJB1–PRKACA* fusion gene is an early and primary driver of fibrolamellar carcinoma and suggests caution in interpreting regional morphological heterogeneity as evidence for a mixed tumor.

We also identified two unusual FISH patterns. These patterns are important because they extend our understanding of the genetic lesions in the *PRKACA* locus and because they potentially could represent an interpretative challenge in clinical care. The first pattern is consistent with a *DNAJB1–PRKACA* fusion gene followed by amplification of this locus, a model which is supported by follow-up qPCR studies. The clinical significance of this finding is unclear because of its rarity, but the tumor was otherwise typical for fibrolamellar carcinoma morphologically and immunohistochemically. The presence of amplification of a fusion gene has been noted in other translocation-associated tumors.^19, 20, 21, 22^ Collection and study of additional fibrolamellar carcinomas with amplification of the *DNAJB1–PRKACA* fusion will be needed to determine the significance of this finding. The second unusual pattern resulted from two genetic events; (i) formation of the fusion gene and (ii) loss of the *PRKACA* locus on the uninvolved allele. The available data does not indicate whether the additional genetic findings in these two cases occurred at the same time of the genetic insult leading to the *DNAJB1–PRKACA* fusion, or whether these two cases indicate additional genetic instability in this region.

In conclusion, *PRKACA* FISH is a powerful tool to confirm the diagnosis of fibrolamellar carcinoma. It provides direct visualization of the key genomic event in fibrolamellar carcinomas and is useful in challenging cases. The FISH assay is highly specific in the context of primary hepatic neoplasia. We propose the best approach for the diagnosis of fibrolamellar carcinoma is based on compatible morphology with either molecular confirmation, or if not available, then confirmation by CK7 and CD68 immunohistochemistry.
